# Antibiotic Prescription Rates After eVisits Versus Office Visits in Primary Care: Observational Study

**DOI:** 10.2196/25473

**Published:** 2021-03-15

**Authors:** Artin Entezarjou, Susanna Calling, Tapomita Bhattacharyya, Veronica Milos Nymberg, Lina Vigren, Ashkan Labaf, Ulf Jakobsson, Patrik Midlöv

**Affiliations:** 1 Center for Primary Health Care Research Department of Clinical Sciences in Malmö/Family Medicine Lund University Malmö Sweden; 2 Capio Go AB Gothenburg Sweden; 3 Department of Clinical Sciences in Lund Lund University Lund Sweden

**Keywords:** telemedicine, antibiotics, streptococcal tonsillitis, cystitis, respiratory tract infection, virtual visit, virtual, eVisit

## Abstract

**Background:**

Direct-to-consumer telemedicine is an increasingly used modality to access primary care. Previous research on assessment using synchronous virtual visits showed mixed results regarding antibiotic prescription rates, and research on assessment using asynchronous chat-based eVisits is lacking.

**Objective:**

The goal of the research was to investigate if eVisit management of sore throat, other respiratory symptoms, or dysuria leads to higher rates of antibiotic prescription compared with usual management using physical office visits.

**Methods:**

Data from 3847 eVisits and 759 office visits for sore throat, dysuria, or respiratory symptoms were acquired from a large private health care provider in Sweden. Data were analyzed to compare antibiotic prescription rates within 3 days, antibiotic type, and diagnoses made. For a subset of sore throat visits (n=160 eVisits, n=125 office visits), Centor criteria data were manually extracted and validated.

**Results:**

Antibiotic prescription rates were lower following eVisits compared with office visits for sore throat (169/798, 21.2%, vs 124/312, 39.7%; *P*<.001) and respiratory symptoms (27/1724, 1.6%, vs 50/251, 19.9%; *P*<.001), while no significant differences were noted comparing eVisits to office visits for dysuria (1016/1325, 76.7%, vs 143/196, 73.0%; *P*=.25). Guideline-recommended antibiotics were prescribed similarly following sore throat eVisits and office visits (163/169, 96.4%, vs 117/124, 94.4%; *P*=.39). eVisits for respiratory symptoms and dysuria were more often prescribed guideline-recommended antibiotics (26/27, 96.3%, vs 37/50, 74.0%; *P*=.02 and 1009/1016, 99.3%, vs 135/143, 94.4%; *P*<.001, respectively). Odds ratios of antibiotic prescription following office visits compared with eVisits after adjusting for age and differences in set diagnoses were 2.94 (95% CI 1.99-4.33), 11.57 (95% CI 5.50-24.32), 1.01 (95% CI 0.66-1.53), for sore throat, respiratory symptoms, and dysuria, respectively.

**Conclusions:**

The use of asynchronous eVisits for the management of sore throat, dysuria, and respiratory symptoms is not associated with an inherent overprescription of antibiotics compared with office visits.

**Trial Registration:**

ClinicalTrials.gov NCT03474887; https://clinicaltrials.gov/ct2/show/NCT03474887

## Introduction

Direct-to-consumer telemedicine is an increasingly used modality to access primary care in Sweden [1]. Such visits can take the form of asynchronous chat-based visits (eVisits) or synchronous video-based visits (virtual visits). While telemedicine has the potential to address many challenges facing primary care [2] and provide an appropriate alternative for minimizing risk of COVID-19 during the current pandemic [3], concerns have been raised regarding overprescription of antibiotics [4] and potential ramifications to increasing widespread antibiotic resistance. Antibiotic resistance is already predicted to cause more deaths than cancer by the year 2050 [5].

Most research has been conducted on data derived from synchronous virtual visits in American health care settings, where antibiotic prescription is historically higher [6], possibly due to a more market-controlled health care system with incentives for high patient satisfaction [7]. Consequently, there have been mixed results regarding antibiotic prescribing following virtual visits in various contexts [4,8-18], with most studies focusing on urinary tract infections (UTIs) and upper respiratory infections. For example, depending on the health care provider, virtual visits for sinusitis have been associated with both higher [14] and lower [10,13] prescriptions rates compared with office visits. Comparisons to urgent care settings often demonstrate lower prescription rates for virtual visits [8,9].

In Sweden, primary care accounts for 61% of medical antibiotic consumption [19], with 30% of consultations concerning infections [20], most commonly upper respiratory tract infections, tonsillitis, and UTIs [20,21]. Guideline adherence in management of these conditions is poor [22-24]. A study on virtual visits reported that 50% to 60% of cases diagnosed with viral pharyngitis had rapid streptococcal antigen testing (RST) performed or no antibiotics prescribed, while 90% of those diagnosed with streptococcal pharyngitis had RST performed or antibiotics prescribed [25]. However, no comparison was made with office visits. There is thus a paucity of literature concerning eVisit investigations, particularly in terms of head-to-head comparisons to office visits, as highlighted by systematic reviews [26,27].

The aim of this study was to investigate if management of sore throat using a specific eVisit platform led to significantly higher rates of antibiotic prescription compared with usual management using office visits. Secondary outcomes include prescription rate following dysuria and other respiratory symptoms, type of antibiotics prescribed, documentation of Centor criteria (used to identify the likelihood of a bacterial infection in adult patients complaining of a sore throat), and set diagnoses.

## Methods

### eVisit Platform

This retrospective cohort study specifically evaluates an eVisit platform (referred to as "the platform" in this paper) used by a major private health care provider. The platform combines automated patient interviewing software with an asynchronous 2-way text-based chat between patient and health care provider. Patients access the platform using their smartphone, tablet, or computer device and choose their chief complaint from a prespecified symptom list. A digital patient history is then taken, allowing the patient to formulate ideas, concerns, and expectations [28] in free-text with the addition of symptom-specific multiple-choice questions based on algorithms. Questions may address UTI symptoms and patient-assessed Centor criteria [29], such as “Do you have any of the following symptoms together with your sore throat?” with choices of “fever,” “swollen lymph nodes on the neck,” “severe pain when swallowing,” “cough,” “white exudates on your tonsils or in the back of your throat” (image not mandatory but recommended). If a patient reports fever, the question “Have you measured your body temperature?” may be asked with choices “no” or “yes” with an option to specify the highest value in degrees Celsius. Photos can be attached when relevant; this is recommended for the management of sore throat. Answers are summarized and presented to a physician for review, and further doctor-patient communication occurs through a text-based conversation, similar to text messaging, with patients and providers messaging each other at their convenience. Physicians can prescribe medications, order laboratory samples, provide patient information, or stay available for up to 72 hours for conservative management. If deemed necessary, the physician can schedule an office visit at a primary health care center of the same health care provider. At the time of the study, the platform used no machine learning technology.

### Setting and Population

As the private health care provider offers both office visits and eVisits using the platform since July 31, 2017, data could be acquired for both visit types. A total of 16 primary health care centers in the county provided office visit data, while national eVisit data was acquired from the online platform. Inclusion criteria were physician visits with a chief complaint of sore throat, cough, cold/flu symptoms, or dysuria as specified by free-form text in the electronic medical record (EMR) as identified by data extraction software (Multimedia Appendix 1). We also included visits with a recorded diagnosis code J030 (streptococcal tonsillitis), J069 (acute upper respiratory infection), or N300 (cystitis). Visits were included if they occurred between March 30, 2016, and March 29, 2017 (office visits only) or March 30, 2018, and March 29, 2019 (eVisits and office visits). Exclusion criteria were patients aged younger than 18 years, male patients with dysuria, and identifiable visits for similar chief complaints in the past 21 days.

### Power Calculation and Recruitment

Previous data from Sweden suggested an antibiotic prescription rate of 59% for patients with sore throat–related diagnoses [20]. Using a binary outcome power calculation with a noninferiority limit of 10%, an alpha level of .05, for 80% power, we estimated needing 300 sore throat visits per group.

**Figure 1:**
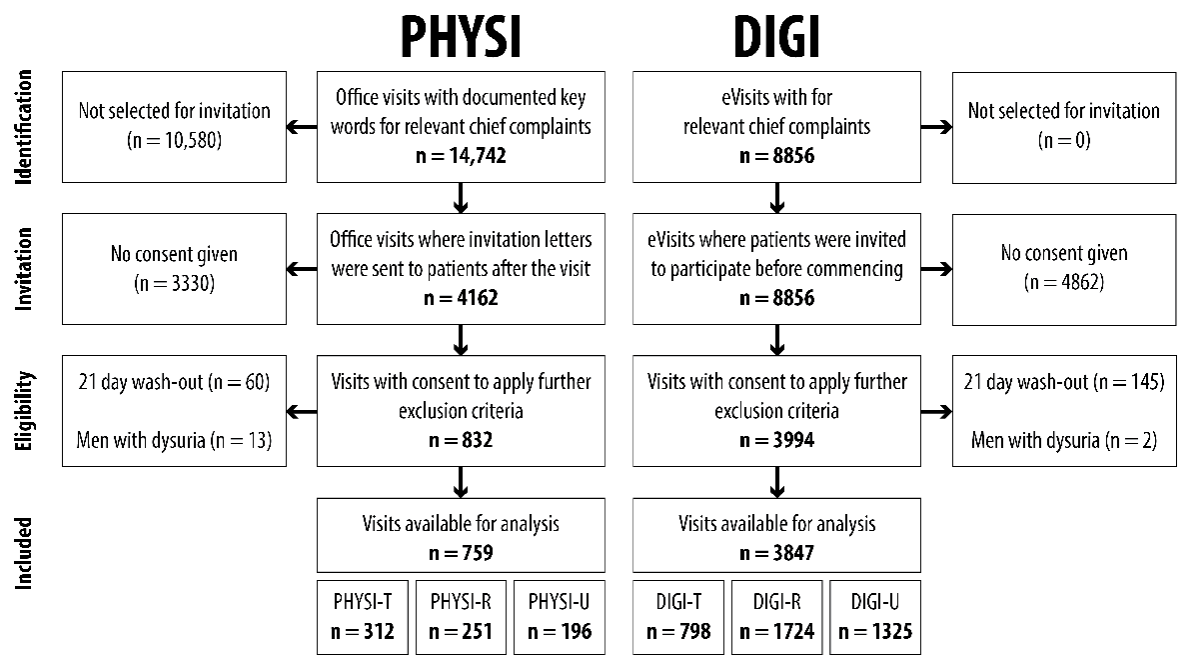
Flowchart of patient recruitment. PHYSI: primary care office visits; DIGI: eVisits; PHYSI-T: office visits with a chief complaint of sore throat; PHYSI-R: office visits with a chief complaint of common cold/influenza or cough; PHYSI-U: office visits with a chief complaint of dysuria; DIGI-T: eVisits with a chief complaint of sore throat; DIGI-R: eVisits with a chief complaint of common cold/influenza or cough; DIGI-U: eVisits with a chief complaint of dysuria.

Digital consent was acquired from eVisit patients at the beginning of the visits and recorded in the EMR. Written consent was acquired from office visit patients, with sore throat patients receiving letters including 2 reminders if no reply was received. Recruitment was completed after consent was acquired from at least 300 sore throat patients in each group. After recruitment, remaining exclusion criteria were applied before analysis commenced (Figure 1).

The health care provider identified 14,742 potential office visits eligible for participation. Letters were then sent to a random selection of 2000 patients with suspected sore throat, 1000 patients with suspected dysuria, and 1000 patients with suspected symptoms of cough, common cold, and influenza, comprising 4162 visits. For office visits with a chief complaint of sore throat (PHYSI-T), 87 patients were recruited after 1 month. An additional 117 patients were recruited after a second letter was sent 2 months later, and an additional 96 patients were recruited 1 month after the third recruitment letter was sent out. A total of 8856 relevant eVisits were identified, from which patients were also invited to participate. In total, we recruited patients from 832 office visits and 3994 eVisits. After exclusion of dysuria visits with male patients and visits within the 21-day washout period, 759 office visits and 3847 eVisits remained for analysis (Figure 1). Office visits were in 99.1% of cases identified via keywords in the free-form text the EMR, while 0.1% (2 sore throat visits, 22 respiratory visits, and 18 dysuria visits) were identified through set diagnoses.

### Diagnostic Criteria and Guideline Adherence

Swedish national guidelines recommend identifying at least 3 Centor criteria (tonsillar exudates, swollen tender anterior cervical nodes, lack of cough, and presence of fever over 38.5° Celsius) prior to ordering an RST [29]. Guidelines recommend that RST should only be performed if the advantages of antibiotic treatment are deemed to outweigh the disadvantages for the individual patient and subsequently recommend penicillin V as first-line treatment [30]. All cases of ordered RST in the presence of Centor criteria were assumed to be due to primary health care physicians deeming the advantages of antibiotic therapy outweighing the disadvantages. In the office visit group, Centor criteria are documented after a physical examination by a physician. For the eVisit group, patients self-assess and report Centor criteria in the automated patient interviewing software [25]. Answers are evaluated by a physician who then chooses which criteria to document in a specified template by, for example, being required to check a box specifying that temperature was above 38.5° Celsius. The physician may choose to document Centor criteria differently from how patients report the criteria depending on what information is acquired during the 2-way patient-provider chat.

### Data Collection

Baseline variables included chief complaint, visiting date, age, and gender. The primary outcome was antibiotic prescription within 3 days following sore throat as the chief complaint, which is similar to previous studies [11,31,32]. Secondary outcomes included antibiotic prescription within 3 days of visits for dysuria and cough/common cold/influenza, type of antibiotic prescribed, documentation of Centor criteria, laboratory tests ordered within 3 days (c-reactive protein [CRP] and RST). Guideline adherence for sore throat patients was also assessed in terms of following indications for antibiotic prescription.

Data extraction software was used to automatically extract data [33,34] with subsets manually validated by reading all free-form text in the EMR and evaluating deviations from automatically extracted data. Variables that were manually evaluated included chief complaint (n=783), Centor criteria (n=285), CRP ordered (n=294), RST ordered (n=284), antibiotic prescription (n=782), and antibiotic type (n=183).

As automatic extraction of free-form text was not possible, Centor criteria for PHYSI-T were manually extracted from a randomly selected subset of the cohort (n=125) while automatically extracted Centor criteria were manually validated for a subset of DIGI-T visits (n=160), resulting in a total of 285 visits with manually validated Centor criteria. Protocols were used for all interpretation of free-form text (Multimedia Appendix 1). For example, free-text documentation stating “fever” was deemed a Centor criterion since only a minority of cases specified temperature in this context.

### Statistical Analyses

Analysis was conducted using SPSS Statistics version 26 (IBM Corporation). A 21-day washout period was applied, excluding past eVisits or office visits for similar chief complaints, similar to previous methods [4]. For this washout, sore throat, cough, and common cold or influenza were all deemed similar chief complaints as they are all respiratory symptoms.

Visits for cough and common cold or influenza, each a separate chief complaint for eVisits, were grouped together for analysis as these chief complaints often result in similar diagnoses, resulting in a total of 6 groups for analysis: sore throat office visit (PHYSI-T) and eVisit (DIGI-T), cough/common cold/influenza office visit (PHYSI-R) and eVisit (DIGI-R), and dysuria office visit (PHYSI-U) and eVisit (DIGI-U). Variables on type of antibiotics prescribed were recategorized to separate antibiotics not commonly recommended by guidelines (Multimedia Appendix 2). For analyses of guideline adherence, manually collected Centor criterion data were dichotomized so that undocumented symptoms were assumed to be absent.

The first diagnosis recorded at each visit was recategorized as UTI, viral upper and lower respiratory tract infection, tonsillitis, and 3 common diagnoses seen as more severe conditions following each of our chosen chief complaints: pneumonia, peritonsillar abscess, and pyelonephritis. Symptom-based codes and nondiagnostic codes were grouped as nonspecific or symptom-based diagnosis and remaining diagnoses were grouped as other (Multimedia Appendix 3). Continuous data were presented with mean and standard deviation and analyzed with Student *t* test, while categorical data were presented with percentage and analyzed with chi-square test.

We hypothesized that there would be no clinically relevant difference in antibiotic prescribing. Hypothesis testing was conducted by comparing office visits to eVisits for each chief complaint. As age and set diagnoses are potential confounding factors for the tendency to prescribe antibiotics, multiple binary logistic regressions were conducted for each chief complaint with antibiotic prescription as the dependent variable and visit type as the independent variable in an enter regression model. The models were then adjusted for age and diagnoses of tonsillitis, viral upper and lower respiratory tract infection, pneumonia, and other diagnoses. eVisits were used as the reference group.

No data were missing for the primary outcome analyses. For secondary outcomes, visits with missing data were compared with visits with valid data for patient age, prescription of antibiotics. and antibiotic choice to test whether data was missing at random. Visits with data missing at random were excluded from the analyses.

Exploratory analyses were conducted for sore throat patients from one county (n=289 for DIGI-T and n=312 for PHYSI-T) where data on Centor criteria and related variables were available for random subsets of the data. Two measures of guideline adherence for sore throat management were explored:

- Proportion of RST performed on properly documented indications (ie, 3 or more documented Centor criteria)
- Proportion of visits diagnosed with tonsillitis that were prescribed antibiotics with a positive RST performed on properly documented indications

### Ethics and Registration

The study was approved by the Swedish Ethical Review Authority (reference number: 2019-00463). Permission to use regional medical record data was also granted (reference number: 062-18). The study was registered at ClinicalTrials.gov [NCT03474887] and reported using a Strengthening the Reporting of Observational Studies in Epidemiology (STROBE) checklist.

### Data Sharing Statement

Data are available to the Department of Clinical Sciences in Malmö at Lund University and can be accessed for a prespecified purpose after approval upon reasonable request.

## Results

### Manual Validation of Data

Manual validation showed high accuracy of extracted data, with 98.7% (773/783) accuracy for antibiotic prescription within 3 days and chief complaint for office visits correctly classified in 98.5% (133/135) for PHYSI-T but less often so for PHYSI-R (212/234, 90.6%) and PHYSI-U (95/103, 92.2%). For PHYSI-U patients, many cases of misclassified patients had lower abdominal pain rather than dysuria.

### Baseline Demographics

For all chief complaints, baseline demographics revealed a significantly higher patient age among office visits compared with eVisits. For both sore throat and respiratory symptoms, around one-third (343/1110, 30.9%, and 721/1975, 36.5%, for sore throat and respiratory symptoms, respectively) of the visits involved male patients, with slightly more men in DIGI-T compared with PHYSI-T (Table 1).

**Table 1:** Baseline demographics.

Chief complaint		Age in years, mean (SD)	*P* value for difference	Sex, male, n (%)	*P* value for difference
**Sore throat (n=1110)**		—^a^	<.001	—	.03
	DIGI-T^b^ (n=798)	35.1 (11.5)	—	262 (32.8)	—
	PHYSI-T^c^ (n=312)	44.5 (17.5)	—	81 (26.0)	—
**Respiratory (n=1975)**		—	<.001	—	.28
	DIGI-R^d^ (n=1724)	42.8 (14.5)	—	637 (36.9)	—
	PHYSI-R^e^ (n=251)	60.0 (16.2)	—	84 (33.5)	—
**Dysuria (n=1521)**		—	<.001	—	—
	DIGI-U^f^ (n=1325)	42.1 (15.4)	—	0 (0.0)	—
	PHYSI-U^g^ (n=196)	60.0 (18.9)	—	0 (0.0)	—

^a^Not applicable.

^b^DIGI-T: eVisits with a chief complaint of sore throat.

^c^PHYSI-T: Office visits with a chief complaint of sore throat.

^d^DIGI-R: eVisits with a chief complaint of common cold/influenza or cough.

^e^PHYSI-R: Office visits with a chief complaint of common cold/influenza or cough.

^f^DIGI-U: eVisits with a chief complaint of dysuria.

^g^PHYSI-U: Office visits with a chief complaint of dysuria.

### Diagnoses

Based on the first diagnosis recorded by the physician, a total of 185 different diagnosis codes were recorded across the entire cohort, with 107 different diagnosis codes for office visits and 98 different diagnosis codes for eVisits.

Nonspecific or symptom-based diagnoses were recorded among 25.3% (973/3847) of eVisits compared with 14.2% (108/759) of office visits, while other diagnoses were recorded for 1.8% (70/3847) of eVisits compared with 19.1% (145/759) of office visits.

Tonsillitis was recorded among 25.8% (206/798) of DIGI-T compared with 33.3% (104/312) of PHYSI-T. Viral upper and lower respiratory diagnoses were recorded among 61.3% (1057/1724) of DIGI-R compared with 48.6% (122/251) of PHYSI-R.

A total of 0.7% (19/2522) recorded diagnoses were for pneumonia across DIGI-T and DIGI-R compared with 2.3% (13/563) across PHYSI-T and PHYSI-R. Peritonsillar abscess was recorded in 0.8% (6/798) of DIGI-T compared with 0.6% (2/312) of PHYSI-T. There was one recorded diagnosis of pyelonephritis among PHYSI-U and none among DIGI-U.

### Antibiotic Prescription

Compared with eVisits, antibiotic prescription within 3 days of the visit was significantly higher for office visits for sore throat and respiratory symptoms. No significant difference in prescription rate was observed for dysuria visits (Table 2).

For respiratory symptoms and dysuria, office visits more often led to the prescription of antibiotics outside of guideline recommendations for tonsillitis and pneumonia, respectively (Table 2).

Odds ratio of antibiotic prescription as the dependent variable following a PHYSI-T visit compared with DIGI-T was 2.46 (95% CI 1.86-3.26; *P*<.001). Adjustment for age and differences in recorded diagnoses had a marginal impact on odds ratios (Table 3).

**Table 2:** Antibiotic-related outcomes. No data were missing among presented variables. See Multimedia Appendix 2 for guideline-recommended antibiotics.

Chief complaint		Antibiotic prescription within 3 days of visit, n (%)	*P* value for difference	Guideline-recommended antibiotics, n (%)	*P* value for difference
**Sore throat (n=1110)**		—^a^	<.001	—	.39
	DIGI-T^b^ (n=798)	169 (21.2)	—	163 (96.4)	—
	PHYSI-T^c^ (n=312)	124 (39.7)	—	117 (94.4)	—
**Respiratory (n=1975)**		—	<.001	—	.02
	DIGI-R^d^ (n=1724)	27 (1.6)	—	26 (96.3)	—
	PHYSI-R^e^ (n=251)	50 (19.9)	—	37 (74.0)	—
**Dysuria (n=1521)**		—	.25	—	<.001
	DIGI-U^f^ (n=1325)	1016 (76.7)	—	1009 (99.3)	—
	PHYSI-U^g^ (n=196)	143 (73.0)	—	135 (94.4)	—

^a^Not applicable.

^b^DIGI-T: eVisits with a chief complaint of sore throat.

^c^PHYSI-T: Office visits with a chief complaint of sore throat.

^d^DIGI-R: eVisits with a chief complaint of common cold/influenza or cough.

^e^PHYSI-R: Office visits with a chief complaint of common cold/influenza or cough.

^f^DIGI-U: eVisits with a chief complaint of dysuria.

^g^PHYSI-U: Office visits with a chief complaint of dysuria.

**Table 3:** Regression models for antibiotic prescription for office visits compared with eVisits.

Chief complaint	Antibiotic prescription within 3 days of office visits vs eVisits, UOR^a^ (95% CI)	*P* value	Antibiotic prescription within 3 days of office visits vs eVisits, AOR^b,c^ (95% CI)	*P* value
Sore throat (n=1110)	2.46 (1.85-3.26)	<.001	2.94 (1.99-4.33)	<.001
Respiratory (n=1975)	15.63 (9.58-25.53)	<.001	11.57 (5.50-24.32)	<.001
Dysuria (n=1521)	0.82 (0.58-1.15)	.25	1.01 (0.66-1.53)	.98

^a^UOR: unadjusted odds ratio.

^b^AOR: adjusted odds ratio.

^c^Each regression model was adjusted for age and diagnoses, tonsillitis, viral upper and lower respiratory tract infection, pneumonia, and other.

### Antibiotic Choice

**Figure 2:**
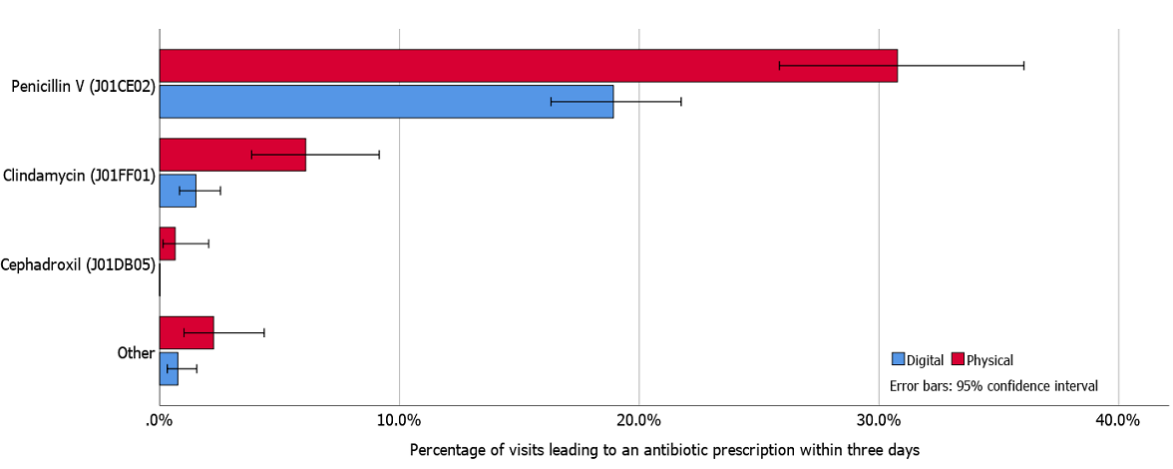
Prescription rates for various antibiotics following chief complaint of sore throat.

**Figure 3:**
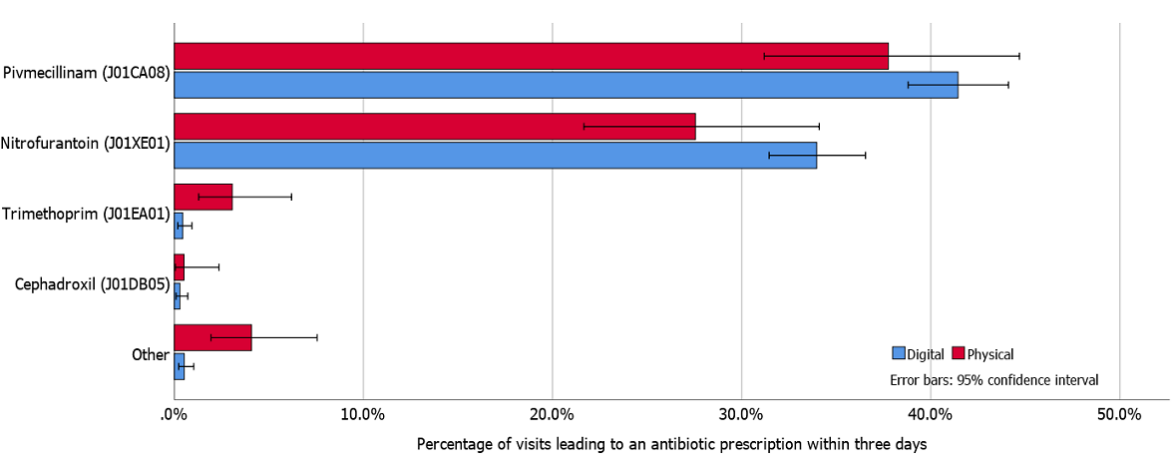
Prescription rates for various antibiotics following chief complaint of dysuria.

**Figure 4:**
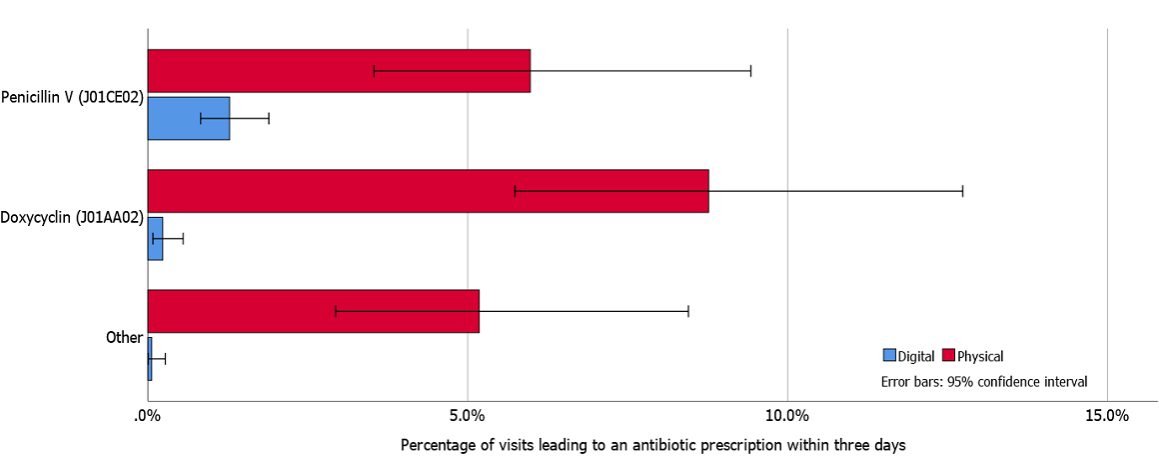
Prescription rates for various antibiotics following chief complaint of respiratory symptoms.

Antibiotic choice was similar for DIGI-T versus PHYSI-T as well as DIGI-U versus PHYSI-U (Figures 2 and 3, respectively). Antibiotic prescriptions following DIGI-R most often led to prescriptions of penicillin V, while PHYSI-R most often led to prescriptions of doxycycline (Figure 4). Penicillin V accounted for 89.3% (151/169) of all prescribed antibiotics among DIGI-T and 77.4% (96/124) of all prescribed antibiotics in PHYSI-T.

Among the 13 sore throat visits included in “Other” (6 DIGI-T, 7 PHYSI-T visits), there was one DIGI-T and one PHYSI-T visit each with UTI diagnoses receiving pivmecillinam, and one PHYSI-T visit with a diagnosis of acute bronchitis receiving doxycycline. Remaining visits had only sore throat–related diagnoses and were followed by prescriptions of doxycycline, erythromycin, and amoxicillin with and without clavulanic acid.

Among the 15 dysuria visits included in “Other” (7 DIGI-U, 8 PHYSI-U visits), 7 PHYSI-U visits led to prescriptions of trimethoprim sulfamethoxazole, methenamine, or ciprofloxacin without a relevant diagnosis to support the prescription given current guidelines, while one PHYSI-U visit led to a diagnosis with pyelonephritis and prescription of ciprofloxacin accordingly. A total of 5 DIGI-U visits had non-specified UTI diagnoses; 3 of these patients were prescribed ciprofloxacin, 1 trimethoprim sulfamethoxazole, and 1 lymecycline. The remaining 2 DIGI-U patients were diagnosed with acute cystitis and prescribed ciprofloxacin.

Among the 14 respiratory visits included in “Other” (1 DIGI-R, 13 PHYSI-R visits), 4 PHYSI-R patients were prescribed amoxicillin, erythromycin, or cefadroxil without a diagnosis supported by guidelines, and 2 PHYSI-R visit patients were prescribed amoxicillin with the diagnosis of pneumonia. A total of 3 PHYSI-R visit patients were diagnosed with concurrent UTIs, 2 of whom were prescribed pivmecillinam and 1 trimethoprim sulfamethoxazole. The remaining PHYSI-R visit patients were diagnosed with chronic obstructive pulmonary disease or acute exacerbation and were prescribed amoxicillin.

### Documentation of Centor Criteria

All 4 Centor criteria were documented for 100% (798/798) of DIGI-T visits and 28% (35/125) of PHYSI-T visits. Documentation did not differ among PHYSI-T visits prescribed antibiotics versus cases not prescribed antibiotics (13/45, 28.9%, versus 22/80, 27.5%, complete documentation, respectively). Specifically, presence or absence of tonsillar exudates, fever, lymphadenopathy, and cough were not documented in 4.8% (6/125), 21.6% (27/125), 26.4% (33/125), and 57.6% (72/125) of PHYSI-T visits, respectively.

Among the subset of sore throat patients from a specific county, there was no significant difference in documented fever between DIGI-T and PHYSI-*t* (116/289, 40.1%, vs 46/125, 36.8%; *P*=.52). PHYSI-T more often had absence of cough (96/125, 76.8%, vs 151/289, 52.2%; *P*<.001). DIGI-T had significantly more documented swollen tender anterior cervical nodes (182/289, 63.0%, vs 39/125, 31.2%; *P*<.001) and tonsillar exudates (136/289, 47.1%, vs 37/125, 29.6%; *P*=.001; Multimedia Appendix 4).

Among manually reviewed cases with documented tonsillar exudates among DIGI-T, 86.6% (116/134) had a photo attached of varying quality in terms of visualizing tonsillar exudates.

### Guideline Adherence for Sore Throat

Exploratory analyses of sore throat visits with Centor criteria data (Multimedia Appendix 5) showed that RST testing was more often performed on properly documented indications in terms of Centor criteria among DIGI-T compared with PHYSI-*t* (105/132, 79.5%, vs 23/70, 32.9%; *P*<.001).

Among visits that were diagnosed with tonsillitis and prescribed antibiotics, there were more cases of positive RSTs performed on properly documented indications among DIGI-T compared with PHYSI-*t* (42/43, 97.7%, vs 8/20, 40.0%; *P*<.001).

## Discussion

### Principal Findings

Rates of antibiotic prescription following eVisits for sore throat, cough, common cold, and influenza were significantly lower than for office visits, while no differences in prescription rates were noted for dysuria. This difference persisted after adjusting for age and set diagnoses.

### Limitations

Results should be interpreted with consideration for several limitations. First, as the groups were not randomized, we were unable to establish causality between visit type and antibiotic prescription rate. However, randomization in this context was not feasible as risk of spillover was high with patients free to seek other forms of care.

We cannot exclude that the lower prescription rate among eVisits reflects a self-selected group with different symptom severity, comorbidity frequency, patient expectations, and time constraints compared with those seeking office care. Differences between physicians working in the digital platform versus in the office setting may be another factor influencing differences in prescription rates.

Differences in recruitment strategy may have impacted the results. During eVisits, patients self-selected their chief complaint, which was then documented and used for recruitment, while office visit physicians chose which symptom to document as the chief complaint. eVisit physicians were not blinded for participation to the study, which may have influenced the outcome.

Regarding sore throat, the results of this study may not apply to countries preferentially using other scoring systems such as the McIsaac score to determine whether to perform an RST [35].

Finally, while we used a 21-day washout period, we cannot exclude that some visits may have been preceded by a visit from another health care provider within the washout period. Across the entire cohort, there were 12 patients who had both an eVisit and an office visit, with the eVisit preceding the office visit in 8 cases. However, visits were always separated by at least 21 days, making conversions clinically unlikely and warranting a novel assessment regarding indications for antibiotic prescription. Our sample size was relatively small but adequate to address the research question.

### Strengths

Despite the above, this study has several strengths. As far as the authors know, this is the first study specifically comparing antibiotic prescription following asynchronous eVisits to office visits outside of the American health care setting. The dataset comes from one of the few health care providers of both eVisits and office visits, thus making the groups more comparable. Using chief complaint as opposed to diagnosis as inclusion criteria means prescription rates may better reflect clinical practice as many clinicians tend to choose diagnoses based on their choice to prescribe antibiotics, regardless of guideline adherence. Using data extraction software ensured reliability of data, and manually reviewing subsets of the data added validity regarding physician assessment and documentation. Findings were robust through logistic regression and several subgroup analyses.

### Interpretation

Beyond potential unidentified confounding factors, the lower antibiotic prescription rate in DIGI-T may reflect the health care providers’ use of a structured documentation platform requiring physicians to actively mark each Centor criterion prior to ordering an RST. It has previously been hypothesized that availability of guidelines may be the driving factor behind improved guideline adherence in virtual visits [8], and decision support systems have previously been shown to improve guideline adherence [16,36].

One must also consider the risk of misdiagnosis with eVisits. There is a risk that the system would lead the physician into a logical conclusion and apparently guideline-coherent decision, increasing the risk of cognitive biases such as confirmation bias, which may not have occurred face-to-face in an office setting.

eVisits may also facilitate physicians to better manage emotionally demanding patients [37], possibly reducing the risk of prescribing antibiotics without proper indications. In addition, eVisits provide a convenient way for physicians to use watchful waiting prior to antibiotic prescription as patients easily can access the chat within 72 hours of a consultation.

 DIGI-T patients are required to visit their nearest primary health care center to take the RST prior to receiving antibiotics, which may create an additional barrier to antibiotic prescription not present in PHYSI-T. These barriers are absent for antibiotic prescription following UTIs, which may explain the similar rates between DIGI-U and PHYSI-U.

As previously mentioned, eVisits involve physician interpretation of patient reported Centor criteria prior to documentation, while office visits involve interpretation of Centor criteria through physical examination prior to documentation. For example, cough may be more correctly reported following eVisits as it is reported much more categorically than when asked in an office setting and interpreted by the physician with a working diagnosis. Conversely, lymphadenopathy may be overreported among eVisits due to self-palpating of cervical myalgia because of a sore throat. The use of patient-reported Centor criteria remains to be validated, prompting some organizations to dissuade management of sore throat patients using eVisits [38]. As future studies are required to validate specific criteria for eVisit diagnosis of streptococcal tonsillitis, this study’s objective was to evaluate adherence to local health care provider protocols.

The seemingly higher proportion of nonspecific or symptom-based diagnoses recorded after eVisits may represent physicians’ reluctancy or inability to make diagnoses through the platform.

A majority of DIGI-T but a minority of PHYSI-T visits with ordered RST had sufficiently documented Centor criteria. Furthermore, a larger proportion of prescribed antibiotics in DIGI-T had a positive RST ordered on correctly documented indications. These findings should be interpreted with caution and warrant replication given their basis in a small random sample of PHYSI-T visits. EMR notes after office visits are often short, and all symptoms may not have been documented in PHYSI-T visits. Thus, PHYSI-T physicians may still adhere to guidelines similarly to DIGI-T, even though this adherence is not documented. It is, however, worth considering that more complete documentation may be a strength of eVisits compared with office visits, regardless of guideline adherence. Antibiotic prescriptions without positive RST following office visits may also be a consequence of general practitioners relying on clinical gaze over laboratory test results [24].

### Comparison With Other Studies

As most studies investigating antibiotic prescribing for visits were selected based on recorded diagnoses such as streptococcal tonsillitis, our findings are not directly comparable as each group in this study contains a range of set diagnoses. However, certain patterns can be noted when the current findings are placed in context.

The finding that antibiotic prescriptions are lower following eVisits for sore throat contrasts with most previous research finding higher prescription rates for virtual visits compared with office visits following diagnosis of pharyngitis [4,15,32], with the exception of one study finding lower prescription rates following diagnosis of nasopharyngitis [32]. Differences in antibiotic prescription in this study persisted after adjusting for age and differences in set diagnoses. However, a retrospective cohort study with a large, matched sample noted no differences in prescription rates for pharyngitis [15]. Given this disparity, the findings in this study warrant replication in a different population.

The finding that DIGI-T more often were prescribed antibiotics per guideline recommendations contrasts with previous studies suggesting overprescription of broad-spectrum antibiotics after virtual visits compared with office visits [15,32]. This may demonstrate that the platform specifically improves guideline adherence through a framework encouraging physicians to reflect on guidelines prior to prescription. This is partially reflected by 100% documentation of Centor criteria, higher than reported from other eVisit platforms [25]. Indeed, previous interventions involving the use of symptom templates demonstrate improved documentation [39].

Regarding respiratory symptoms, the lower prescription rate noted in this study is in line with most research on virtual visits finding similar or lower prescription rates for bronchitis and acute respiratory infections compared with office visits [4,15,18,32,40], although some studies found higher broad-spectrum prescription rates for bronchitis [18,32].

For dysuria, previous research noted higher prescription rates following virtual visits [4] as well as eVisits [11] compared with office visits. However, a recent study on management of UTIs using asynchronous eVisits found no differences in antibiotic prescription rates. Our findings support this latter finding and the use of telemedicine for the management of uncomplicated UTIs [12]. This also suggests that eVisits and virtual visits may differently impact antibiotic prescribing.

### Conclusions

The use of asynchronous eVisits for the management of sore throat, dysuria, or respiratory symptoms does not appear to lead to an inherent overprescription of antibiotics compared with office visits, even after considering differences in age and recorded diagnoses. Antibiotic prescriptions do not seem to deviate from guidelines more often than usual management using office visits. Findings support the use of structured eVisits in the context of a platform with an infrastructure encouraging guideline adherence. Future research is needed to confirm the findings of this study and validate the use of Centor criteria or another set of criteria to use for differential diagnosis and treatment of conditions related to sore throat in the eVisit setting.

## Appendix

Multimedia Appendix 1 Protocol for interpretation of free-form text for validation of data. In uncertain cases, dialogue occurred with a family medicine specialist in order to determine if symptoms should be deemed present or absent. As not all visits were manually validated, all visits were included in the analysis.

Multimedia Appendix 2 Anatomic therapeutic chemical classification codes for recategorization of prescriptions according to current Swedish guideline recommendations.

Multimedia Appendix 3 Recategorization of diagnoses.

Multimedia Appendix 4 Centor criteria for a subset of sore throat patients from a specific county. Denominators based on available data (unavailable data is missing at random).

Multimedia Appendix 5 Secondary outcomes related to guideline adherence for a subset of sore throat visits from a specific county. Denominators vary due to missing data (unavailable data is missing at random).

## Abbreviations

CRP: c-reactive protein
EMR: electronic medical record
eVisit: asynchronous chat-based visit
PHYSI-T: office visit with a chief complaint of sore throat
RST: rapid streptococcal antigen testing
STROBE: Strengthening the Reporting of Observational Studies in Epidemiology
UTI: urinary tract infection
