# Figure Correction: Antibiotic Prescription Rates After eVisits Versus Office Visits in Primary Care: Observational Study

**DOI:** 10.2196/34529

**Published:** 2021-11-26

**Authors:** Artin Entezarjou, Susanna Calling, Tapomita Bhattacharyya, Veronica Milos Nymberg, Lina Vigren, Ashkan Labaf, Ulf Jakobsson, Patrik Midlöv

**Affiliations:** 1 Center for Primary Health Care Research Department of Clinical Sciences in Malmö/Family Medicine Lund University Malmö Sweden; 2 Capio Go AB Gothenburg Sweden; 3 Department of Clinical Sciences in Lund Lund University Lund Sweden

In “Antibiotic Prescription Rates After eVisits Versus Office Visits in Primary Care: Observational Study” (JMIR Med Inform 2021;9(3):e25473) the authors noted one error.

In the originally published paper, the flowchart of patient recruitment (Figure 1) contained incorrect exclusion criteria which are irrelevant for the current publication and thus had incorrect numbers. The figure also did not include the acronyms PHYSI, DIGI, PHYSI-T, PHYSI-R, PHYSI-U, DIGI-T, DIGI-R, and DIGI-U as specified in the figure caption. The figure has been replaced with the correct version, which includes all previously stated acronyms. The headings “Office visits” and “eVisits” have been replaced with “PHYSI” and “DIGI”, respectively. “Patients available for analysis” has been replaced with “Visits available for analysis.” Exclusion criteria now correctly include only “21 day wash-out” and “Men with dysuria.” The updated version of Figure 1 that will appear in the corrected manuscript is displayed below. The originally published version of Figure 1 can be found in Multimedia Appendix 1.

**Figure 1:**
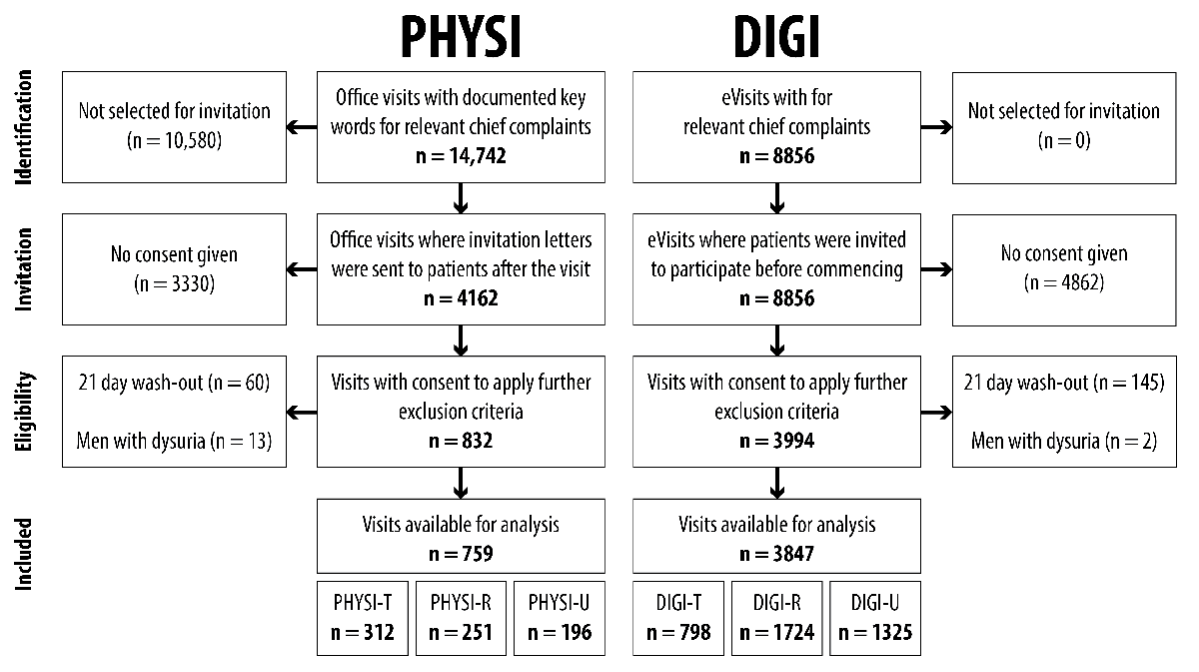
Flowchart of patient recruitment. PHYSI: primary care office visits; DIGI: eVisits; PHYSI-T: office visits with a chief complaint of sore throat; PHYSI-R: office visits with a chief complaint of common cold/influenza or cough; PHYSI-U: office visits with a chief complaint of dysuria; DIGI-T: eVisits with a chief complaint of sore throat; DIGI-R: eVisits with a chief complaint of common cold/influenza or cough; DIGI-U: eVisits with a chief complaint of dysuria.

The correction will appear in the online version of the paper on the JMIR Publications website on November 26, 2021, together with the publication of this correction notice. Because this was made after submission to PubMed, PubMed Central, and other full-text repositories, the corrected article has also been resubmitted to those repositories.

## Appendix

Multimedia Appendix 1 Originally published Figure 1.

