# Supplementary material for: Antibiotic Prescription Rates After eVisits Versus Office Visits in Primary Care: Observational Study
Source: JMIR Med Inform. 2021 Mar 15;9(3):e25473. doi: 10.2196/25473 (PMC8077790; doi:10.2196/25473)
Supplement: Multimedia Appendix 1 [file medinform_v9i3e25473_app1.docx]

*Appendix 1: Protocol for interpretation of free-form text for validation of data. In uncertain cases, dialogue occurred with a family medicine specialist (SC) in order to determine if symptoms should be deemed present or absent. As not all visits were manually validated, all visits were included in the analysis.*

|  | Free-form text interpreted as variable being present | Free-form text interpreted as variable being absent |
| --- | --- | --- |
|  |  |  |
| **Sore throat** | Text indicating pain from the inner pharyngeal cavity | Text indicating unpleasant sensations outside of the pharyngeal cavity, including the oral cavity.  Dysphagia |
| **Dysuria** | Text indicating a painful or unpleasant sensation in conjunction with micturition.  Text indicating urgency.  Text indicating an increased frequency of micturition. | Lower abdominal pain.  Isolated flank/back pain. |
| **Respiratory symptoms** | Text indicating infection-related cough, influenza or common cold. | Non-infectious and allergic cough. |
| **Fever** | “Fever”, with or without specified temperature, at any time point since symptom debut.  “Temperature over 38.5 degrees” (despite not specified in ordered labs) | “Feeling of fever”  “Feeling warm”  “Possibly fever”  “Sub-febrile”  “Non-febrile”  “Unclear if fever”  “Likely fever” |
| **Tonsillar exudates** | “White coatings over the entire palate”  “Suggestive of coatings” | “Redness in throat”  “Red tonsils”  “Swollen tonsils”  “Red macules in palate roof”  “Swollen, red tonsils”  “Enlarged tonsils”  “Mucous membranes free from irritation”  “Oral cavity and pharynx: 0”  “Oral cavity and pharynx without remark” |
| **Lymphadenopathy** | Lymph nodes in jaw angle described as tender or swollen, either bilaterally or unilaterally:  “Lymph nodes slightly enlarged right”  “Lymph nodes swollen on the neck”  “Tender adenitis”  “Submandibular lymph nodes”  “Lymph nodes behind sternocleidomastoids  “Lymph node package on the neck”  “Adenitis and tenderness over the tonsils”  “Bilaterally tender on the neck” | “Clinically like tonsillitis”  “Large tonsils upon palpation”  “Throat without remark”  “Lymph nodes without remark”  “Swollen on the neck” |
| **Absence of cough** | Any mention of cough not being present.  “No common cold symptoms” | Any mention of current cough |
