# Supplementary material for: Antibiotic Prescription Rates After eVisits Versus Office Visits in Primary Care: Observational Study
Source: JMIR Med Inform. 2021 Mar 15;9(3):e25473. doi: 10.2196/25473 (PMC8077790; doi:10.2196/25473)
Supplement: Multimedia Appendix 2 [file medinform_v9i3e25473_app2.docx]

*Appendix 2: Recategorization of prescriptions according to current Swedish guideline recommendations. Anatomic therapeutic chemical classification codes in parentheses.*

|  | Sore throat | Dysuria | Respiratory |
| --- | --- | --- | --- |
|  |  |  |  |
| **Categorized as within guideline recommendations*** | Penicillin V (J01CE02)  Clindamycin (J01FF01)  Cefadroxil (J01DB05) | Pivmecillinam (J01CA08)  Nitrofurantoin (J01XE01)  Trimethoprim (J01EA01)  Cefadroxil (J01DB05) | Penicillin V (J01CE02)  Doxycycline (J01AA02) |
| **Categorized as outside of guideline recommendations*** | Ciprofloxacin (J01MA02) Erythromycin (J01FA01)  Lymecycline (J01AA04)  Methenamine (J01XX05)  Doxycycline (J01AA02)  Nitrofurantoin (J01XE01)  Pivmecillinam (J01CA08) Amoxicillin (J01CA04)  Amoxicillin with clavulanic acid (J01CR02) Trimethoprim sulfamethoxazole (J01EE01) | Penicillin V (J01CE02)  Ciprofloxacin (J01MA02)  Erythromycin (J01FA01)  Lymecycline (J01AA04)  Methenamine (J01XX05)  Doxycycline (J01AA02) Amoxicillin (J01CA04) Amoxicillin with clavulanic acid (J01CR02)  Trimethoprim sulfamethoxazole (J01EE01) | Ciprofloxacin (J01MA02)  Erythromycin (J01FA01)  Cefadroxil (J01DB05)  Lymecycline (J01AA04)  Methenamine (J01XX05)  Nitrofurantoin (J01XE01)  Pivmecillinam (J01CA08) Amoxicillin (J01CA04) Amoxicillin with clavulanic acid (J01CR02) Trimethoprim sulfamethoxazole (J01EE01) |

** Guideline recommendations for treatment of tonsillitis, uncomplicated cystitis in women and pneumonia were used, for sore throat, dysuria and respiratory symptoms, respectively.*
