# Supplementary material for: Antibiotic Prescription Rates After eVisits Versus Office Visits in Primary Care: Observational Study
Source: JMIR Med Inform. 2021 Mar 15;9(3):e25473. doi: 10.2196/25473 (PMC8077790; doi:10.2196/25473)
Supplement: Multimedia Appendix 3 [file medinform_v9i3e25473_app3.docx]

*Appendix 3: Recategorization of diagnoses*

| Categorization | Diagnosis code (ICD-10 and KSH97-P) |
| --- | --- |
|  |  |
| **Viral upper and lower respiratory tract infection** | J069, J06P, J02, J209, J111, J22P, J019, Y555, J029, J11P, J068, B27, J01, J04, J009, J06-, J229, J010, J118, J312, J370, J399, J42P |
| **Urinary tract infection** | N300, N30P, N309, N308, N329, N34, N341, N390, N39 |
| **Tonsillitis** | J030, J03, J038, J039 |
| **Pneumonia** | J189, J18P, J159 |
| **Peritonsillar abscess** | J36, J369 |
| **Pyelonephritis** | N12P |
| **Nonspecific or symptom-based diagnosis** | R059, R070, R398, B34P, R509, R05, B349, Z038, H920, R35, R060, R300, R539, Z711, Z039, A49P, F51, G933, J02-, K62, R040, R042, R329, R104, R319, R529, K590, R30, Y553, L299, L29P, N398 , R065, R391, R490, R520, R599, B99, I888, I899, K137, K309, K30P, L298, R119, R196, R221, R238, R30-, R309, R359, R39., R39B, R52, Z03, Z048, Z719, no diagnosis |
| **Other** | E03, J459, I10, H100, J441, B370, E66P, E78, H109, N394, J45P, K21, N952, B373, B379, E73, F17, F419, H60, H612, B49P, E118, H609, H65, J301, J449, J450, K64, L309, M255, A090, A16P, A379, A600, B009, B309, C53, C61, C64P, C85P, C91, D25, D64P, D730, D868, E061, E079, E109, E119, E14P, E271, F172, F31, F411, F430, F439, G259, G439, G473, H010, H103, H660, H663, H730, H811, H939, I109, I110, I25P, I48, I89P, J302, J45-, K050, K120, K50, K573, K80, L82, L859, M069, M13P, N393, N811, T784 |
