# Supplementary material for: Antibiotic Prescription Rates After eVisits Versus Office Visits in Primary Care: Observational Study
Source: JMIR Med Inform. 2021 Mar 15;9(3):e25473. doi: 10.2196/25473 (PMC8077790; doi:10.2196/25473)
Supplement: Multimedia Appendix 4 [file medinform_v9i3e25473_app4.docx]

*Appendix 4:* *Centor criteria for a subset of sore throat patients from a specific county. Denominators based on available data (unavailable data is missing at random).*

|  | DIGI-T visits from specific county  (n = 289) | PHYSI-T visits from specific county (n = 312) | *P* value for difference |
| --- | --- | --- | --- |
|  |  |  |  |
| **Fever**  **(%)** | 116/289  (40.1%) | 46/125  (36.8%) | .52 |
| **Tonsillar exudates (%)** | 136/289  (47.1%) | 37/125  (29.6%) | .001 |
| **Lymphadenopathy (%)** | 182/289  (63.0%) | 39/125  (31.2%) | < .001 |
| **Absence of cough (%)** | 151/289  (52.2%) | 96/125  (76.8%) | < .001 |
