# Supplementary material for: Antibiotic Prescription Rates After eVisits Versus Office Visits in Primary Care: Observational Study
Source: JMIR Med Inform. 2021 Mar 15;9(3):e25473. doi: 10.2196/25473 (PMC8077790; doi:10.2196/25473)
Supplement: Multimedia Appendix 5 [file medinform_v9i3e25473_app5.docx]

*Appendix 5: Secondary outcomes related to guideline-adherence for a subset of sore throat visits from a specific county. Denominators vary due to missing data (unavailable data is missing at random).*

|  | DIGI-T visits from specific county  (n = 289) | PHYSI-T visits from specific county (n = 312) | *P* value for difference |
| --- | --- | --- | --- |
|  |  |  |  |
| **CRP taken within three days (%)** | 68/289  (23.5%) | 137/312  (43.9%) | † |
| **RST taken within three days (%)** | 132/289  (45.7%) | 171/298  (57.4%) | † |
| **RST taken within three days and three or more documented Centor criteria (%)** | 105/132  (79.5%) | 23/70  (32.9%) | < .001 |
| **Diagnosed with tonsillitis (%)** | 81/289  (28.0%) | 104/312  (33.3%) | † |
| **Diagnosed with tonsillitis and prescribed antibiotics within three days (%)** | 51/81  (63.0%) | 86/104  (82.7%) | † |
| **Diagnosed with tonsillitis and prescribed antibiotics within three days and RST taken (%)** | 44/51  (86.3%) | 64/78  (82.1%) | † |
| **Diagnosed with tonsillitis and prescribed antibiotics within three days and RST taken with positive RST result (%)** | 43/44  (97.7%) | 53/64  (82.8%) | † |
| **Diagnosed with tonsillitis and prescribed antibiotics within three days and RST taken with positive RST result and three or more documented Centor criteria**  **(%)** | 42/43  (97.7%) | 8/20  (40.0%) | < .001 |

*†: No hypothesis testing performed.*
