# Supplementary figures and images for: Figure Correction: Antibiotic Prescription Rates After eVisits Versus Office Visits in Primary Care: Observational Study
Source: JMIR Med Inform. 2021 Nov 26;9(11):e34529. doi: 10.2196/34529 (PMC8726763; doi:10.2196/34529)

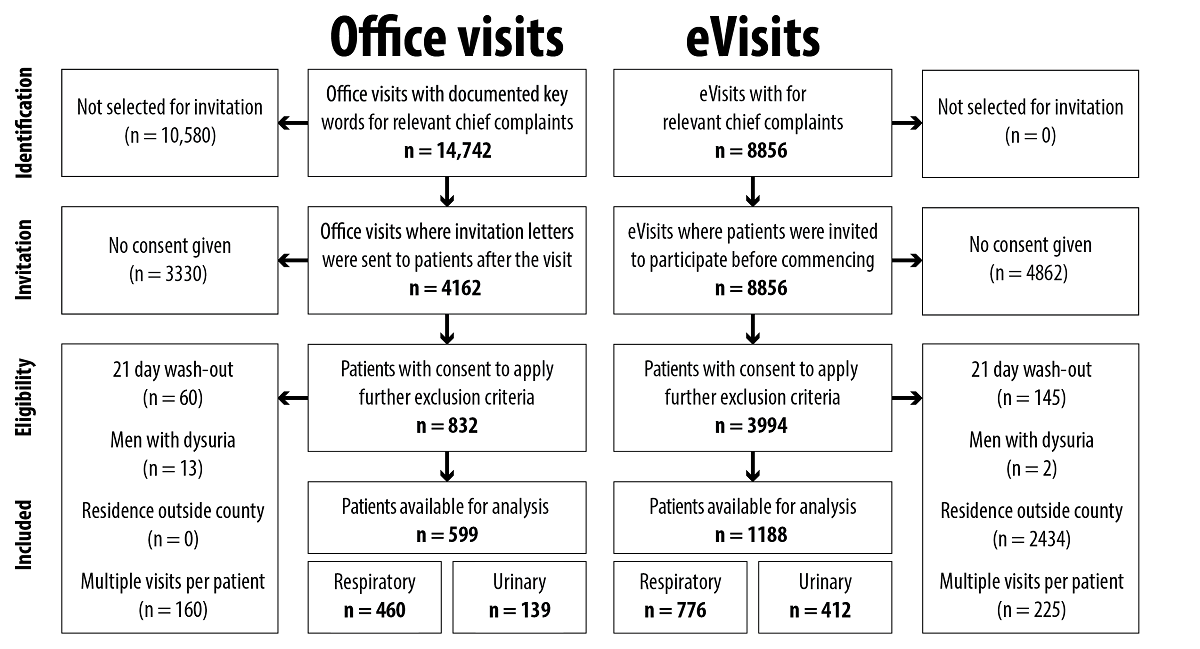

Supplement: Multimedia Appendix 1 [file medinform_v9i11e34529_app1.png]
